# The Child Illness and Resilience Program (CHiRP): a study protocol of a stepped care intervention to improve the resilience and wellbeing of families living with childhood chronic illness

**DOI:** 10.1186/2050-7283-2-5

**Published:** 2014-03-11

**Authors:** Katrina M Hamall, Todd R Heard, Kerry J Inder, Katherine M McGill, Frances Kay-Lambkin

**Affiliations:** Hunter Institute of Mental Health, Hunter New England Local Health District, Newcastle, Australia; School of Medicine and Public Health, University of Newcastle, PO Box 833, Newcastle, NSW 2300 Australia; Hunter New England Population Health, Hunter New England Local Health District, Newcastle, Australia; NHMRC Centre for Research Excellence in Mental Health and Substance Use, Centre for Translational Neuroscience and Mental Health, University of Newcastle, Newcastle, Australia; National Drug and Alcohol Research Centre, University of New South Wales, Sydney, Australia; Centre for Translational Neuroscience and Mental Health, University of Newcastle, Newcastle, Australia

**Keywords:** Family resilience, Childhood chronic illness, Family intervention, Evaluation, Parental wellbeing, Family functioning, Social support

## Abstract

**Background:**

Families of children living with chronic illness are more vulnerable to mental health problems, however this can be ameliorated by a family’s resilience. The Child Illness and Resilience Program (CHiRP) will develop and evaluate a parent-focussed family intervention designed to increase the resilience and wellbeing of families living with childhood chronic illness.

**Methods/Design:**

The study will be conducted in an Australian regional paediatric hospital and will use a stepped care intervention that increases in intensity according to parental distress. All parents of children discharged from the hospital will receive a family resilience and wellbeing factsheet (Step 1). Parents of children attending selected outpatient clinics will receive a family resilience and wellbeing activity booklet (Step 2). Parents who receive the booklet and report psychological distress at three-month follow-up will be randomised to participate in a family resilience information support group or waitlist control (Step 3). The Step 3 control group will provide data to compare the relative effectiveness of the booklet intervention alone versus the booklet combined with the group intervention for distressed parents. These participants will then receive the information support group intervention. All parents in Step 2 and 3 will complete baseline, post-intervention and six month follow up assessments. The primary outcomes of the study will be changes in scores between baseline and follow-up assessments on measures of constructs of family resilience, including parental wellbeing, family functioning, family beliefs and perceived social support. Qualitative feedback regarding the utility and acceptability of the different intervention components will also be collected.

**Discussion:**

It is hypothesised that participation in the CHiRP intervention will be associated with positive changes in the key outcome measures. If effective, CHiRP will provide an opportunity for the health sector to deliver a standardised stepped care mental health promotion intervention to families living with childhood chronic illness.

**Trial registration:**

Australian clinical Trials Registry ACTRN 12613000844741

Universal Trial Number (UTN): 1111-1142-8829

## Background

Almost two in five families in Australia live with a child with a chronic illness (AIHW 2012). A chronic illness is one that is prolonged in duration, does not often resolve spontaneously and is rarely cured completely (Dowrick et al. 2005). It includes illnesses such as asthma, diabetes, cystic fibrosis, musculoskeletal and inflammatory disorders and gastroenterological disorders. Better survival rates mean the number of children living with chronic illnesses are increasing and consequently, there is a growing need to better understand the family impacts of caring for a child with a chronic illness (O'Brien et al. 2009; Scholten et al. 2011).

Children and young people who live with a chronic illness experience a range of persistent stressors that can increase their risk of developing mental health problems (Barlow & Ellard 2006; Cadman et al. 1987; Combs-Orme et al. 2002; Lavigne & Faier-Routman 1992; Mikkelsson & Sourander 1997; Sein 2001). For example, these children may need to manage symptoms that introduce physical and lifestyle limitations, including restricted participation in school and sport, as well as treatment effects and the impact of incorporating ongoing treatment into daily activities. These factors may limit spontaneity, create challenges for social relationships, and generate concerns about the future (Scholten et al. 2011; Gannoni & Shute 2010; Taylor et al. 2008).

The social and emotional impacts of living with childhood chronic illness are not confined to the child or young person themselves; they are shared with other family members (Barlow & Ellard 2006; Bauman et al. 1997; Raina et al. 2005). Parents of children and young people living with a chronic illness experience increased levels of stress due to intensified demands of their care and support role, financial pressures, and strains on their relationships with immediate and extended family members (Gannoni & Shute 2010; Raina et al. 2005; Barlow et al. 2006; Melnyk et al. 2001; Tong et al. 2008). Siblings are at increased risk of anxiety and intense emotions, experience confusion and difficulty communicating about the illness, may feel overlooked, and can experience negative changes to family and social functioning as a result of their sibling’s illness (O'Brien et al. 2009; Gannoni & Shute 2010; Bellin & Kovacs 2006; Besier et al. 2010; Strohm 2008).

While the experience of living with childhood chronic illness can increase vulnerability to mental ill-health, there is a growing body of research that suggests despite increased risk, some families are able to positively manage the impacts of the illness on their lives (Bellin & Kovacs 2006). Such families utilise their experience of managing the illness to develop stronger relationships, coping skills, refocus priorities, recognise opportunities and adapt to the situation so they achieve a sense of functioning just as well as ‘healthy’ families (Gannoni & Shute 2010; Huygen et al. 2000). The ability to maintain healthy family functioning, adapt to stressful life events and develop strengths and skills as a result, reflects a family’s resilience (Rolland & Walsh 2006).

A family resilience framework can guide mental health promotion interventions. Strategies for promoting wellbeing can target the processes and factors involved in coping, adjustment and resilience (Walsh 2002). Theoretical models of family resilience describe a dynamic process through which families are able to adjust in healthy ways to change, cope with stressors, build on strengths, draw on protective factors and modify family functioning to support optimal adaptation in the face of adverse experiences (Rolland & Walsh 2006; Walsh 2002; Benzies & Mychasiuk 2009; McCubbin & McCubbin 1996). Resilience-based mental health promotion interventions can assist families to identify their strengths, recognise the protective factors and resources they can utilise and build on within their family and the environment, and provide opportunities to practice specific strategies to improve coping and family functioning (Padesky & Mooney 2012).

Interventions aimed at improving the mental health and wellbeing of families living with childhood illness provide evidence that helping families to identify positive coping skills (Barrera et al. 2002; Sahler et al. 2013; Sansom-Daly et al. 2012; Scholten et al. 2013), enhance family functioning (Hocking & Lochman 2005; Jerram et al. 2005; Lobato & Kao 2002; Loding et al. 2008) and access resources including social support (Chernoff et al. 2002; Merkel & Wright 2012; Monaghan et al. 2011; Stewart et al. 2011) result in positive patient and family outcomes. There is evidence that interventions targeting parents have positive outcomes for the child and family, given that parental wellbeing and family functioning has a significant impact on the child’s health outcomes and coping with the illness (Sein 2001; Raina et al. 2005; Tong et al. 2008; Scholten et al. 2013; Hocking & Lochman 2005; Eccleston et al. 2012; Patterson & Geber 1991; Peek & Melnyk 2010) and sibling adjustment (Sahler et al. 2013; Drotar & Crawford 1985). The literature also suggests that the efficacy and acceptability of parent based interventions is enhanced when conducted in a group setting, as the group provides the additional benefit of opportunities for exchange of information, shared experience and mutual support for parents experiencing child-illness related stress (Chernoff et al. 2002; Merkel & Wright 2012; McCarthy & Sebaugh 2011; Turner et al. 2001).

Although existing research supports the efficacy of interventions to improve the mental health and wellbeing of families living with chronic illness, there are a number of limitations to the existing evidence. Published reviews report a focus on improving disease management (e.g. enhancing treatment adherence, reducing or managing symptoms) with fewer studies addressing psycho-social outcomes or using a health promotion approach (Peek & Melnyk 2010; Barlow & Ellard 2004; Glasscoe & Quittner 2008). Furthermore, previous studies have used insufficiently validated outcome measures, short follow up periods, small sample sizes, non-standardized intervention delivery or provided inadequate detail of intervention content (Eccleston et al. 2012; Barlow & Ellard 2004; Glasscoe & Quittner 2008; Pai et al. 2006). Previous interventions have also been limited by the absence of a theoretical framework for a project’s design and reliance on delivery by mental health professionals within clinical settings (Peek & Melnyk 2010; Marsac et al. 2012). Thus, there is a need for studies that develop and evaluate interventions designed to improve the mental health of families living with childhood chronic illness that address these limitations.

This paper describes the methodology for the Child Illness and Resilience program (CHiRP). The CHiRP program is a stepped-care mental health promotion intervention guided by family resilience theory designed to support families who care for a child or young person with chronic illness. The intervention resources will assist families to identify existing strengths and provide strategies that target key protective factors and processes that enhance family resilience, such as family functioning, coping skills and utilising resources including social support.

### Study aim and hypotheses

This study aims to improve the resilience and wellbeing of families caring for a child living with chronic illness. Specifically, the objectives of the study are to: 1) improve the capacity of health services to provide family resilience information and strategies to families; 2) determine if the CHiRP resources are acceptable to parents; 3) increase parents knowledge and confidence to implement strategies to improve their family resilience; 4) promote parental help seeking behaviours and 5) improve the resilience and psychological wellbeing of families by providing a stepped care intervention to parents that increases in intensity according to the family’s situation. It is hypothesised that a significant positive improvement in outcome measures of constructs indicating family resilience (parental wellbeing, family functioning, family beliefs, and perceived social support) will be observed between baseline and follow up assessments as a result of receiving a family resilience self-directed booklet. Furthermore, for parents who are experiencing psychological distress, it is hypothesised that participation in a parent information support group (in addition to receiving the booklet) will result in greater improvement on the same outcome measures. It is further anticipated that participants will report that the intervention content and delivery style is acceptable.

## Methods/Design

### Study design and setting

**Figure 1:**
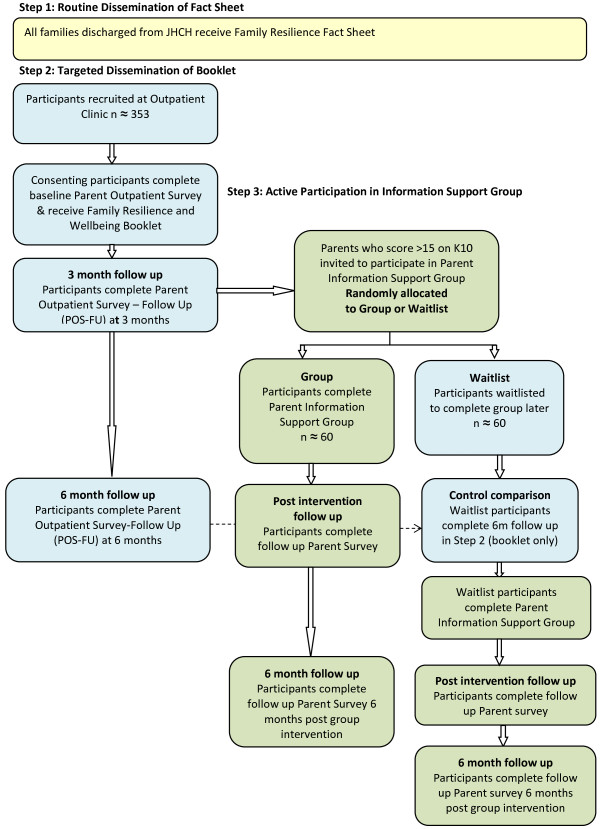
**Flow diagram of CHiRP research design.**

The setting is a paediatric hospital in regional New South Wales that provides healthcare services to approximately 16,675 children per year who reside in the Newcastle, Hunter and New England regions (Australia). The intervention uses a stepped care design, such that increasing levels of intervention support are provided to families living with childhood chronic illness based on the level of parental distress. Step one involves the routine dissemination of a Family Resilience and Wellbeing Fact Sheet using single group assignment. Step two involves the targeted dissemination of a parent-focussed Family Resilience and Wellbeing information and activity booklet and will employ a repeated measures pre-post design. Step three involves the active participation of parents in a Family Resilience and Wellbeing Information Support Group and will employ a randomised waitlist control design. The dissemination strategy and research design for each step of the intervention is outlined in Figure 1.

### Participants and recruitment

Note, throughout this study, “parents” are defined as the adults who identify as being the primary carer of the child attending the outpatient clinic (this could include the biological parent, guardian or other adult family member) and “child” refers to a person less than 18 years of age.

#### Step 1 routine dissemination: fact sheet intervention

All parents who have a child who is discharged from the paediatric hospital will receive a Family Resilience and Wellbeing Fact Sheet. This will be automatically printed with paediatric discharge summaries and routinely disseminated alongside normal clinical practice. This component of the intervention will ensure all families who have a child discharged from the wards of the paediatric hospital, regardless of the reason for admission, will receive standardised brief family resilience and wellbeing support information. There will be no direct data collection from this group.

#### Step 2 targeted dissemination: booklet intervention

All parents who have a child who attends one of four selected outpatient clinics at the paediatric hospital will be considered eligible to participate in this stage of the intervention. The outpatient clinics at the paediatric hospital were selected for inclusion based on patient occupancy, illness presentations and clinic attendance rates. The clinics include Cystic Fibrosis, Diabetes, Gastroenterology and Rheumatology. These clinics provide a service predominantly to children living with a diagnosed chronic illness. Clinics operate once a week on a rotating basis and provide appointments to approximately 350 children per month in total.

Parents with children attending these selected outpatient clinics will be invited to participate in the program via a mailed invitation that includes the participant information statement, consent form and questionnaire. This will be delivered a minimum of two weeks prior to their child’s clinic appointment. The booklet will be provided to participants once the research team receives the completed questionnaire.

#### Step 3 active dissemination: parent information support group intervention

Participants who report psychological distress at the 3-month follow up of Step 2 will be considered eligible to participate in Step 3 of the intervention. Psychological distress will be defined according to cut off scores on the Kessler Psychological Distress Scale (K10) (Kessler et al. 2003). There is some evidence that support groups are of optimal benefit for this portion of the target population (Turner et al. 2001). All parents reporting a K10 score above 15 (Slade et al. 2011) will be eligible for the group. Eligible parents will be contacted by phone by a member of the CHiRP research team and invited to attend an Information Support Group. Participants who agree to attend the Information Support Group will be randomly allocated to the group or waitlist condition using standard statistical computer software. Researchers will not be blinded to group assignment. Participants will be notified by phone and letter of the date of group commencement.

### Data collection procedures

#### Step 1 routine dissemination: fact sheet intervention

Routine collection of discharge data from the paediatric hospital over the intervention period (using the NSW Health Admitted Patient Data Collection) will provide an estimate of the number of families who have received the Fact Sheet.

#### Step 2 targeted dissemination: booklet intervention

As per Figure 1, baseline data for Step 2 will be collected through the CHiRP Parent Outpatient Survey. This will be completed by all participants who participate in Step 2, prior to receiving the Booklet intervention. The questionnaire will take approximately 20 minutes to complete and a random identification code will be generated to maintain participant confidentiality. Participants will complete the questionnaire at home or while waiting for the clinic appointment, and it will be available to complete in hard copy or online. Baseline data collection for Step 2 will occur from September 2013 to March 2014.

Follow up data will be collected from all participants in Step 2 at three and six months after completing the baseline assessment and receiving the Booklet intervention. On both follow up occasions, to minimise attrition, participants will receive a prompt letter advising that follow up will occur. Participants will be contacted by phone approximately two weeks after receiving the prompt letter and be given the option to complete the questionnaire over the phone with a member of the CHiRP team, be sent the questionnaire to complete in hard copy (and return it with a reply paid envelope), or complete the questionnaire online. Data collection for the three-month follow up assessment of Step 2 will occur between January – June 2014. Data collection for the 6-month follow up assessment is anticipated to occur between April - September 2014.

#### Step 3 active dissemination: parent information support group intervention

The data from the three-month follow-up assessment for Step 2 will constitute the baseline (or pre-group) data for Step 3. Participants in Step 3 will also complete an additional questionnaire at the first group session regarding their expectations of the group and what mental health support they might already be receiving. Medical records for the child of parents in Step 3 will be reviewed to record any hospital-based nursing or allied health staff involvement with the family.

On completion of the Information Support group, participants will be provided with the follow up questionnaire as part of the last group session. Follow up assessment of the group intervention will occur between April – September 2014. Participants in the waitlist control group will be completing the 6-month follow up assessment from baseline in Step 2 of the study at this time. This will provide the control comparison data to assess the additional impact of the group intervention, compared to receiving the booklet alone. The waitlist control group will then proceed to participate in the group intervention. All participants in Step 3 will be scheduled to complete a final follow up (single-group) assessment six months after they attended the group. This will provide repeated measures pre-post data regarding the longer-term efficacy of the group intervention. Participants will receive a prompt letter regarding six month follow up and approximately two weeks later a phone call, giving them the option to complete the questionnaire over the phone, online or receive a hard copy in the mail. Six-month follow up of participants in Step 3 is anticipated to occur between October 2014 – March 2015.

### Measures

#### Client characteristics

The baseline questionnaire will collect data on age, gender, ethnicity, education level, employment status and perceived financial adversity (FaHCSIA 2007). Questions also ask about health-related help-seeking in the last three months, their child’s chronic illness, health of other family members and consultations regarding their own mental health or wellbeing. Respondents will also rate their general wellbeing, physical and mental health, ability to perform tasks, their relationships with others and involvement in the community.

#### Outcome measures

##### Parental wellbeing

The Kessler Psychological Distress Scale (K10) is a 10-item screening tool to assess distress and the likelihood of a mental disorder in the responding individual (Kessler et al. 2003; Slade et al. 2011; Andrews & Slade 2001). The K10 uses a five-point rating scale asking how often in the last four weeks participants have experienced symptoms pertaining to nervousness, agitation, psychological fatigue and depression. Higher scores indicate higher levels of distress. The K10 has established validity and acceptable reliability (Andrews & Slade 2001; Centre for Population Studies in Epidemiology 2002).

##### Family functioning

The McMasters Family Assessment Device (FAD) will be used to measure family functioning (Ryan et al. 2012). The FAD identifies strengths as well as limitations in family functioning across seven dimensions including problem solving, communication, roles, affective responsiveness, affective involvement, behaviour control and general functioning (Ryan et al. 2012). Statements describe various aspects of family functioning and respondents indicate agreement on a four-point scale, with lower scores indicating better family functioning. The FAD has adequate internal and test retest reliability (Epstein et al. 1983; Miller et al. 1985) and appropriate construct and criterion validity (Miller et al. 1985; Kabacoff et al. 1990).

##### Social connectedness

The Medical Outcomes Study Social Support Survey (MOSSSS) assesses perceived availability of functional support (Sherbourne & Stewart 1991). Respondents indicate how often each kind of support is available if they need it on a five-point scale. The 19 items represent four dimensions of support: emotional/informational support, tangible support, affectionate support and positive social interaction. Higher scores indicate higher perceived support. The scale has demonstrated good internal reliability and construct validity (Sherbourne & Stewart 1991).

##### Family belief systems

Family beliefs are a construct that contributes to family resilience (Rolland & Walsh 2006; Walsh 2002; Walsh 2003). In a study designed to measure Walsh’s Family Resilience Framework, Sixbey (Sixbey 2008) developed a measure which included subscales of ‘maintaining a positive outlook’ and ‘ability to make meaning of adversity’ (Walsh 2003; Sixbey 2008). Despite limited psychometric validity (Sixbey 2008), the items were deemed appropriate for inclusion in the Parent Outpatient Survey. Data analysis will include examination of factor loadings to examine the validity of this construct in contributing to CHiRP participants’ resilience (see below).

#### Other

A follow up parent survey will measure the impact of Step 2, the booklet intervention. The questionnaire will be administered at three and six months after baseline. It will include the outcome measures as well as questions about changes to the child’s diagnosis in the past three months, questions about the acceptability of the booklet and questions to illicit participant’s self-reported knowledge, understanding and confidence to implement strategies. A follow up parent survey will also be used to assess Step 3. The questionnaire will be administered immediately following the group intervention and then six months later. The questionnaire will include the outcome measures as well as questions regarding perceived utility and acceptability of the group content and format.

### Intervention

The CHiRP intervention content and style has been informed by a literature review of interventions aimed at improving the wellbeing of families living with childhood chronic illness (Incledon et al. 2013; Hiscock et al. 2012), and consultation with the target group (including parents, children with chronic illness and siblings of children with chronic illness) and allied health and medical staff at the paediatric hospital. The intervention promotes the importance of family routines, relationship building, parenting skills, self-care, cognitive restructuring, communication, problem-solving skills and accessing social support.

#### Step 1 routine dissemination: fact sheet intervention

The Family Resilience and Wellbeing Fact Sheet is a two-sided, A4 document, produced in a style concordant with existing hospital fact sheets. The Fact Sheet provides brief psycho-education and practical resilience building strategies for families. Contact information is provided for a small number of relevant community organisations.

#### Step 2 targeted dissemination: booklet intervention

The Family Resilience and Wellbeing booklet, entitled “Strong Parents, Resilient Families” is a 73-page, A4 sized, colour illustrated spiral bound booklet. The booklet uses a parent-focussed, strengths-based, cognitive behavioural approach to promoting family resilience (Padesky & Mooney 2012). The content encourages parents to identify family strengths, develop an understanding of the characteristics of a resilient family, identify goals for skills and strengths building and practice specific strategies to improve family resilience. Parents are encouraged to complete the resilience building activities together with family members. The booklet also contains a comprehensive list of community services for families and children living with chronic illness. Table 1 provides an overview of the booklet content.

**Table 1:** **Overview of intervention content: strong parents, resilient families booklet**

Module	Content
Introduction	• Definition of resilience
	• Simple model of family resilience
	• Instructions and summary of booklet content
	• Case study
1. Building a strong family	• Getting to know my family activity
	• Shared lives, shared milestones timeline activity
	• Family support inventory activity
2. Building a resilient family	• Resilient families factsheet
	• Family strengths checklist activity
	• Maintain routines activity
	• Looking after yourself and each other activities
	• Family communication factsheet and worksheet
	• Hints and tips for spending time together as a family
	• Positive thinking worksheets
	• Problem solving factsheet and worksheet
	• Respectful relationships factsheet/worksheet
	• Parenting tips factsheet and parenting strategies worksheet
	• Understanding an illness factsheet and self-management activity
3. Preparing for the future	• List of family support services

#### Step 3 active dissemination: parent information support group intervention

The Parent Information Support Group is an education, support and skills development group program directed toward parents experiencing psychological distress. The group program content will be based on the “Strong Parents, Resilient Families” booklet and will provide the additional benefit of an opportunity for discussion and practice of the strategies within a facilitated, interactive peer-supported environment.

Parents managing childhood chronic illness have significant demands on their time and participation in face-to-face groups can be low and attrition rates high (Stewart et al. 2012). In light of this, the information support group will combine face-to-face delivery with interactive online delivery strategies. The intervention will be conducted over six weeks. In week one, participants will attend a full day session (4 hours + 1 hour lunch) during which they will have the opportunity to connect with other parents, share their experience and interact with the facilitators to engage with the material in the “Strong Parents, Resilient Families” booklet. This session will include up to 15 participants and will be run by two trained facilitators with a background in psychology or allied health. Over weeks two to five, material pertaining to family resilience strategies will be posted once a week onto an online forum by a research team member who will act as moderator of the site. Participants will be encouraged to read the material, engage in the activity with their family, and post their comments for group online discussion. In week six, a final session will provide an opportunity for parents to meet face-to-face to discuss their experiences, review their participation in the program and complete the evaluation. A facilitator manual detailing the content, structure and format of each session and online material will maximise standardised delivery of the intervention content. Collaboration with outpatient clinic staff (e.g. Clinical Nurse Consultants or Social Workers) and carer support services will allow for delivery of the program to occur with the support of existing service providers.

### Ethics approval

CHiRP has been approved by the Hunter New England Human Research Ethics Committee (Ref No.13/03/20/4.06) and the University of Newcastle Human Research Ethics Committee (Ref No. H-2013-0157).

### Sample size

In Step 1, it is expected that the Fact Sheet will be provided to 1050 parents of children discharged from paediatric hospital services based on an estimate of inpatient numbers over a 12-month period in 2011. There will be no direct data collection from this group.

A sample size calculation was conducted to guide recruitment for Step 2, the booklet intervention. Using mean estimates based on normative scores (Andrews & Slade 2001; Kabacoff et al. 1990; Sherbourne & Stewart 1991; Sixbey 2008) and a hypothesised effect size of 0.2 as a result of receiving the family resilience and wellbeing booklet, a minimum sample of 98 participants at the three-month follow up assessment will have sufficient power (0.8) to detect a significant difference between mean scores (using alpha 0.05) in a one-sample comparison of baseline and three month follow-up data. A larger sample size will be recruited, however, to allow for attrition and sufficient numbers of participants eligible for Step 3.

In Step 3, the Parent Information Support Group intervention, using a randomised waitlist control design, the sample size will be divided randomly and equally between the group condition and the waitlist control condition. A sample size calculation used mean estimates based on a sample of Australian patients diagnosed with a mental disorder (Kohn et al. 2012) using the K10 and normative scores for the other outcome measures of interest. Effect sizes of 0.5 and 0.3 were hypothesised as a result of receiving the booklet intervention and participating in the information support group intervention or being in the control group, respectively. A sample size of approximately 120 (60 per group) participants at the three month follow up assessment will allow detection of a significant difference between mean scores using an alpha level of 0.05 in a two-sample comparison of the group condition and control condition on the outcome measures, with a power of at least 0.8.

### Statistical analyses

#### Step 1 routine dissemination: fact sheet intervention

Descriptive statistics only will be used to describe the reach of the factsheet; and whether participants in Step 2 and Step 3 recall receiving the factsheet.

#### Step 2 targeted dissemination: booklet intervention

Using oneway analyses of variance (ANOVA), the relationship between demographic data (e.g. gender, age categories, ethnicity, socioeconomic level, help-seeking behaviour categories) and baseline outcome measures will be explored.

The impact of the booklet intervention will be examined using analysis of covariance (ANCOVA) to compare changes in mean scores from baseline to three-month follow-up on the outcome measures for all participants in Step 2 (n ≈ 353). To test for sustained change, a second analysis of the Step 2 participants will use a repeated measures mixed model to compare mean scores on outcome measures at baseline, three and six month follow-up for the participants who did *not* move into Step 3 (the Information Support Group Intervention phase). Further analyses will adjust for baseline characteristics to control for any confounding effects.

#### Step 3 active dissemination: information support group intervention

Initial analyses will use demographic data to compare the characteristics of participants who agree to participate in Step 3 compared to those who decline to participate in this step of the project (despite meeting eligibility criteria, i.e. K10 > 15). Differences in responses according to demographic variables on the construct measures of interest (K10, FAD, family belief systems and MOSSSS) will also be analysed using oneway ANOVAs.

To examine the additional impact of the information support group intervention, relative to the wait-list control group (who will have only received the booklet intervention), an analysis of covariance will examine changes in mean scores on the outcome measures from baseline to post-intervention follow-up according to Group (n ≈ 60) versus Waitlist (n ≈ 60) allocation. To account for participant deviations from the protocol (such as dropout or failure to sufficiently participate in the group intervention), two analyses will be conducted. Data will be analysed using the intention-to-treat principle, which will assume all participants randomised to the intervention or waitlist group completed the study as per the research design and a per-protocol analysis will include only those participants who participate in a minimum of two-thirds of the intervention (Porta et al. 2007). Following post-intervention follow-up data collection, the waitlist group will complete the information support group intervention, and complete the post-intervention follow up. All group-based intervention participants will complete a six-month follow-up assessment. Thus, to test for sustained change, a second analysis will compare mean scores at baseline, post-intervention and six-month follow up on the same outcome measures for all participants in Step 3 of the intervention (n ≈ 120). There will be no treatment allocation variable in this second analysis. Further analyses will adjust for baseline characteristics to control for any confounding effects.

Additional analyses will identify risk and protective factors on each construct measure (for example, scoring above 15 on the K10) (Slade et al. 2011) at baseline for Group and Waitlist participants, and include comparisons for participants who agree to participate in Step 3 of the intervention and attend the Information Support group, compared to those who decline to participate in this step of the project, despite meeting eligibility criteria.

## Discussion

CHiRP (Steps 1-3) aims to promote the psychological wellbeing and resilience of families of children and young people living with a chronic illness. This paper has provided an overview of the methodology to be employed to implement and evaluate a stepped care family resilience and wellbeing intervention.

The CHiRP intervention is guided by family resilience theory and identifies parents’ capacity to implement change to support optimal outcomes for the whole family. Utilising a behaviour change model (Padesky & Mooney 2012) parents will be encouraged to recognise family strengths, coping skills and resources and be provided with the opportunity to develop skills and knowledge that promote resilience.

The effectiveness of CHiRP will be measured using standardised, psychometrically validated instruments of relevant constructs, i.e. parental wellbeing, family functioning, family beliefs and social support, which collectively provide a measure of family resilience. Positive changes in outcome measures will indicate improved family wellbeing and resilience. Renzaho et al. (2013) have demonstrated a relationship between family functioning and parental psychological distress. It is expected that improvements in family functioning, including the dimensions of problem solving, communication, roles and behaviour will reflect adaptation and coping with chronic illness (Walsh 2002; McCubbin & McCubbin 1996) and will be associated with improved parental wellbeing. Changes on the family beliefs scales will reflect the process in resilience where families make meaning of adversity and maintain a positive outlook. Positive change on the social support measure will indicate improved perception of the availability of social support, a protective factor contributing to family resilience (Walsh 2002; Benzies & Mychasiuk 2009; McCubbin & McCubbin 1996). Analysis will also be able to contribute to the development of a psychometrically valid and reliable method for measuring family resilience.

CHiRP will employ a stepped care design to meet the demands of scientific rigour as well as conform to the pragmatic considerations involved in implementing research in a busy paediatric hospital setting. The dissemination of the Fact Sheet in Step 1, as part of routine paediatric discharge procedures, maximises the opportunity for families of hospitalised children (regardless of reason for admission) to receive brief, standardised information regarding ways to promote family resilience. The use of a single group pre-post design in Step 2, while potentially limiting the generalizability of outcomes for the booklet intervention, corresponds to the practicalities involved in implementing mental health promotion practices in the busy outpatient clinic environment (Merkel & Wright 2012). The use of a randomised waitlist control group in Step 3 ensures that the impact of the Information Support group can be rigorously evaluated, while allowing all consenting parents who are experiencing psychological distress to receive the highest level of intensity of the CHiRP intervention.

CHiRP meets an identified need for mental health promotion and prevention interventions that enhance family resilience and wellbeing in families of children with chronic illness who are more vulnerable to mental health issues. Through provision of the Family Resilience Fact Sheet and the Strong Parents, Resilient Families workbook, this study will provide information and strategies in a format that families can access and work through independently, without relying on intensive mental health or medical professional delivery. These family interventions are designed to empower parents to help the whole family cope better by learning new strategies and interacting with each other (Marsac et al. 2012). Further, through provision of the Information Support Group this study will provide additional support to parents reporting the highest levels of distress. If effective, CHiRP will increase the capacity of health services to provide standardised family resilience information and strategies to families living with a child with chronic illness.
